# A close association of freedom from pain, migraine-related functional disability, and other outcomes: results of a post hoc analysis of randomized lasmiditan studies SAMURAI and SPARTAN

**DOI:** 10.1186/s10194-021-01303-w

**Published:** 2021-08-28

**Authors:** Richard B. Lipton, Simin K. Baygani, Stewart J. Tepper, John H. Krege, Raghavendra Vasudeva, Eric M. Pearlman, Paula M. Hauck, Li Shen Loo

**Affiliations:** 1 Albert Einstein College of Medicine, Bronx, NY USA; 2 Eli Lilly and Company, Corporate Center, Indianapolis, IN 46285 USA; 3 Geisel School of Medicine, Dartmouth College, Hanover, NH USA

**Keywords:** Lasmiditan, Migraine, Pain freedom, Pain relief, Most bothersome symptom (MBS), Functional disability, Efficacy endpoints

## Abstract

**Background:**

While pain freedom at 2 h is a key primary outcome for current trials for acute treatment of migraine, the relationship between the degree of head pain and other efficacy measures at 2 h has rarely been explored. Following lasmiditan treatment of a migraine attack with moderate or severe head pain, we contrast those who achieve pain freedom with those who achieve mild pain but not pain freedom 2 h post dosing.

**Methods:**

Patient-level data were pooled across studies and treatment arms from two Phase 3 trials comparing lasmiditan and placebo, SAMURAI and SPARTAN. This post hoc analysis assessed freedom from the most bothersome symptom (MBS), freedom from migraine-related functional disability (disability), and improved patient global impression of change (PGIC) in patients who achieved 2 h pain freedom compared to those who experienced 2 h mild pain. Mild pain differs from pain relief which is defined as either mild pain or pain freedom.

**Results:**

Patients who achieved 2 h pain freedom (*N* = 913), in comparison with those with 2 h mild pain (*N* = 864), were significantly more likely to experience MBS freedom (91.9% vs. 44.9%), disability freedom (87.1% and 13.4%), and improved PGIC (86.5% and 31.5%) (*p* < 0.001 for all combinations). In addition, more patients who were pain free experienced both 2 h MBS freedom and 2 h functional disability freedom (83.6%) compared to those with mild pain (10.8%; *p* < 0.001). The proportion of patients with pain freedom who did not achieve either MBS or disability freedom (4.6%) was lower than in patients with mild pain (52.4%). Lastly, 55.2% of patients experienced mild pain before disability freedom compared to 72.1% who experienced pain freedom and disability freedom at the same time.

**Conclusions:**

This study demonstrated that, at 2 h post treatment, patients who were pain free were more likely to achieve other outcomes including freedom from their MBS, freedom from migraine-related functional disability, and improved PGIC compared to those with mild pain, confirming that 2 h pain freedom is more robustly associated with other clinical outcomes than the 2 h mild pain endpoint.

**Trial Registration:**

SAMURAI (NCT02439320); SPARTAN (NCT02605174).

**Supplementary Information:**

The online version contains supplementary material available at 10.1186/s10194-021-01303-w.

## Background

Previously, the pain-related endpoint in migraine clinical studies accepted by regulatory bodies was 2-h pain relief (also called headache response or headache relief); it was defined as a decrease in head pain intensity from moderate or severe at the time of treatment to mild or no pain 2 h post-dose [1]. The recommended primary endpoint for acute treatment of migraine attacks was revised by the International Headache Society (IHS) and Food Drug Administration (FDA) guidelines to pain freedom at 2 h post-dose for the following reasons: 1) placebo rates for pain relief at 2 h are variable and may exceed 50% [1], 2) the pain relief endpoint defines some patients with incomplete response as achieving success, and 3) judgements about mild pain may be more subjective than judgements about no pain [2]. According to the FDA guidance, a drug effect on headache pain alone is not sufficient to claim efficacy because migraine is a complex disorder. Thus, the current recommended approach is to assess the effects of acute treatments on pain freedom along with freedom from the patients’ self-identified most bothersome symptom (MBS) 2 h post-dose, chosen from photophobia, phonophobia, or nausea [3].

The relationship of pain relief and pain freedom to other acute treatment outcomes, including freedom from associated symptoms and disability as well as satisfaction with treatment has not been well explored.

Therefore, we pooled data from the two lasmiditan randomly assigned, controlled, Phase 3 studies, SAMURAI and SPARTAN, to analyze the concordance of clinical efficacy outcomes. Rather than comparing pain relief (which by definition, includes some patients with pain freedom as well as patients with mild pain) with pain freedom, we compared 3 mutually exclusive head pain outcome categories 2 h post treatment (no pain, mild pain, and moderate or severe pain) to determine the relationship of pain outcome with other clinical outcomes at 2 h post-dose.

## Methods

### Study design

Pooled patient-level data from double-blind, multicenter Phase 3 trials, SAMURAI (NCT02439320) and SPARTAN (NCT02605174), were included in this post hoc analysis. Details of the original study designs, randomization, and inclusion and exclusion criteria were reported previously [4, 5].

The studies adhered to the International Conference on Harmonization Good Clinical Practice guidelines and local regulatory requirements. The protocols were approved by an independent ethics committee or an institutional review board at each study site. Patients provided written informed consent for study participation.

The baseline parameters at the time of dosing for inclusion in this analysis were moderate or severe pain, a self-identified MBS (nausea, phonophobia, or photophobia), and some degree of migraine-related functional disability (functional disability).

### Efficacy endpoints

Patients recorded headache pain intensity (none, mild, moderate, or severe) and whether they were experiencing nausea, phonophobia, photophobia, or vomiting at baseline and 0.5, 1, 1.5, 2, 3, 4, 24, and 48 h post-dose in an electronic diary.

Patient responses at 2 h were categorized into three mutually exclusive head pain outcome subgroups: pain freedom (reduction of headache pain severity from moderate or severe at baseline to none); improvement to mild pain (reduction from moderate or severe head pain at baseline to mild); and continued moderate/severe pain.

The pain efficacy outcome responses were compared with other clinically relevant efficacy outcomes of freedom from MBS (defined as the absence of MBS) and freedom from migraine-related functional disability. Functional disability was assessed at all time points using a 4-point ordinal scale with the question “How much is your migraine interfering with your normal activities?”. Response options were “not at all”, “mild interference”, “marked interference”, or “need complete bed rest”. Functional disability freedom was defined as having “Not at all” recorded. We also assessed patient global impression of change (PGIC). PGIC is an integrated measure of drug tolerability and efficacy that captures the patient’s view of improvement or decline in overall well-being after treatment [6]. PGIC was assessed with the question “How do you feel after taking study medication?” with responses recorded using a 7-point Likert scale, ranging from “very much better” to “very much worse”. Patients who reported “much better” or “very much better” were considered to have improved with treatment.

### Statistical analyses

All outcome combination and sequence assessments were evaluated using individual patient data. Patient-level data were pooled across studies and treatment arms (50 mg [SPARTAN only], 100 mg, 200 mg lasmiditan, and placebo) and analyzed by treatment groups. The modified intent-to-treat (mITT) population was used and included patients who took the randomly assigned treatment within 4 h of migraine onset and provided post-treatment data on headache severity or symptoms. Outcomes reported through 2 h post-dose were included in this analysis; patients were considered to fail outcomes at each time point if they used rescue medication at or before that time point.

For comparisons between outcome combinations, *p*-values were computed from a two-sided test from a logistic regression model with study, head pain outcome group, and background use of medication to reduce the frequency of migraine attacks as covariates. Firth’s penalized likelihood approach was used to address potential population separation and small sample modelling concerns. Odd ratios were calculated only in cases with sufficient patients (≥10) to yield reasonable estimates. A *p*-value of < 0.05 was considered to indicate a statistically significant difference.

We analyzed the associations between outcome pairs (outcome A and outcome B) in a 2-by-2 matrix. The odds of two positive outcomes (outcome A-positive and outcome B-positive) in an outcome pair was tested using a Cochran-Mantel-Haenszel test with stratification by study.

For the subset of patients who achieved both a successful pain outcome and MBS freedom or functional disability freedom at 2 h, we examined the order of occurrence for those outcomes: outcome A before outcome B, or outcome B before outcome A, or both outcomes at the same assessment time point. The numerators were the number of patients within each category defined by order of outcome occurrence, and the denominator was the number of patients who experienced dual outcome by 2 h post-dose. Patients were indicated as unsustained if they moved into and then out of the dual positive outcome group within the interval from dosing to 2 h post-dose.

## Results

### Patient demographics and baseline characteristics

The pooled study sample included 913 individuals who were pain free, 864 individuals who improved to mild pain, and 1052 individuals with moderate or severe pain 2-h after treatment. Patient demographics were generally similar across the 2-h head pain outcome groups. A higher proportion of patients in the group that continued to have moderate/severe pain used migraine preventive medications, had severe headache pain, and required complete bed rest at baseline (Tables 1-2).

**Table 1:** Patient baseline demographics by head pain outcome group at 2 h post-dose

Parameter	Pain freedom (***N*** = 913)	Improved to mild pain (***N*** = 864)	Continued moderate/severe pain (***N*** = 1052)
Age, mean (SD)	41.9 (12.9)	41.8 (12.1)	42.6 (11.8)
Female, n (%)	780 (85.4)	745 (86.2)	885 (84.1)
White, n (%)	711 (78.0)	734 (85.0)	908 (86.3)
Body mass index, mean (SD)	30.3 (7.8)	30.4 (10.6)	29.9 (7.8)
Family history of coronary artery disease, n (%)	277 (30.3)	251 (29.1)	346 (32.9)
Duration of migraine history years, mean (SD)	17.2 (12.6)	19.0 (12.8)	20.0 (12.8)
Average migraine attacks/month in past 3 months, mean (SD)	5.1 (1.6)	5.2 (1.9)	5.4 (2.1)
Use of migraine preventive medication^a^, n (%)	155 (17.0)	186 (21.5)	252 (24.0)

^a^Based on the question “Is the subject currently using medications to reduce the frequency of migraine episodes?” asked during randomization

Notes: Data from the total population (all treatment arms combined). *N* = number of patients in the analysis population with 2-h pain response

SD: standard deviation

**Table 2:** Baseline migraine characteristics by head pain outcome group at 2 h post-dose

Baseline migraine characteristic	Pain freedom (***N*** = 913)	Improved to mild pain (***N*** = 864)	Continued moderate/severe pain (***N*** = 1052)
Time to dosing hours, mean (SD)	1.0 (1.7)	1.29 (1.69)	1.25 (1.56)
Pain severity, n (%)			
Severe	230 (25.2)	170 (19.7)	390 (37.1)
Moderate	683 (74.8)	694 (80.3)	662 (62.9)
Migraine-associated symptoms, n (%)			
Photophobia	726 (79.5)	713 (82.5)	903 (85.8)
Phonophobia	600 (65.7)	576 (66.7)	740 (70.3)
Nausea	384 (42.1)	395 (45.7)	556 (52.9)
Migraine-related functional disability^a^, n (%)			
Need complete bed rest	131 (14.3)	112 (13.0)	245 (23.3)
Marked interference	489 (53.6)	521 (60.3)	612 (58.2)
Mild interference	293 (32.1)	231 (26.7)	195 (18.5)

^a^Functional disability was assessed with the question “How much is your migraine interfering with your normal activities?”. Response options were “not at all”, “mild interference”, “marked interference”, or “need complete bed rest”

Notes: Data from the total population (all treatment arms combined)

SD: standard deviation

### Concordance of pain outcomes with freedom from MBS, freedom from functional disability, and improved PGIC at 2 h

At 2 h, most patients with pain freedom (*N* = 913) also experienced MBS freedom (91.9%) or functional disability freedom (87.1%). In contrast, patients with mild pain (*N* = 864), or continued moderate/severe pain (*N* = 1052) showed lower rates of MBS freedom (44.9% and 10.3%, respectively; *p* < 0.001 vs group with pain freedom for both) or functional disability freedom (13.4% and 1.1%, respectively; *p* < 0.001 for both). Similarly, most patients (86.5%) who experienced pain freedom also reported improved PGIC compared to patients with mild pain or moderate/severe pain (31.5% and 1.7%, respectively; *p* < 0.001 for both) (Table 3).

**Table 3:** Coexistence of positive outcome(s) with head pain outcome at 2 h

Proportion with additional outcomes at 2 h		Pain freedom (*N* = 913)	Improved to mild pain (*N* = 864)	Continued moderate/ severe pain (*N* = 1052)
Number of outcomes	Outcome(s)			
1	MBS freedom, n (%)	839 (91.9)	388 (44.9)*	108 (10.3)*
	Functional disability freedom^a^, n (%)	795 (87.1)	116 (13.4)*	12 (1.1)*
	PGIC (much better/very much better)^b^, n (%)	790 (86.5)	272 (31.5)*	18 (1.7)*
2	Both MBS freedom and disability freedom^a^, n (%)	763 (83.6)	93 (10.8)*	3 (0.3)
3	MBS freedom, disability freedom^a^, and PGIC (much better/very much better)^b^, n (%)	683 (74.8)	64 (7.4)*	2 (0.2)
Neither MBS nor disability freedom^a^ achieved, n (%)		42 (4.6)	453 (52.4)*	935 (88.9)*
Neither MBS freedom, disability freedom^a^, nor PGIC (much better/very much better) ^b^ achieved, n (%)		16 (1.8)	356 (41.2)*	925 (87.9)*

*p < 0.001 versus group with pain freedom at 2 h. *P*-values were only calculated from odds ratios if the number of patients in each specific category was ≥10

^a^Functional disability was assessed with the question “How much is your migraine interfering with your normal activities?” Response options were “not at all”, “mild interference”, “marked interference”, or “need complete bed rest”. Functional disability freedom was defined as having “Not at all” recorded at 2 h. Patients who recorded “Not at all” at the time of dosing were excluded from the analysis

^b^PGIC was assessed with the question “How do you feel after taking study medication?” with responses recorded using a 7-point Likert scale, ranging from “very much better” to “very much worse”. Patients who reported “much better” or “very much better” were considered to have improved with treatment

Notes: Data from the total population (all treatment arms combined). *p*-values were generated from a two-sided test from a logistic regression model with study, head pain outcome group, and background use of medication to reduce the frequency of migraine as covariates. Firth’s penalized likelihood approach was used to address issues of quasi-complete separation

MBS: most bothersome symptom; PGIC: patient global impression of change

The likelihood of patients experiencing two or more positive outcomes, from among MBS freedom, functional disability freedom or improved PGIC, for each of the head pain outcome groups was also examined. Patients with pain freedom at 2 h experienced both MBS freedom and functional disability freedom outcomes together more frequently (83.6%) compared to those with mild pain (10.8%; *p* < 0.001) or continued moderate/severe pain (0.3%). Furthermore, patients were much more likely to experience all three positive outcomes (MBS freedom, functional disability freedom, and improved PGIC) if they experienced pain freedom (74.8%) than if the pain improved to mild (7.4%; *p* < 0.001) or if the pain continued to be moderate/severe (0.2%). As expected, very few patients experienced pain freedom without achieving MBS freedom or functional disability freedom (4.6%, compared to mild pain or continued moderate/severe pain in 52.4% and 88.9%, respectively; *p* < 0.001) (Table 3). For patients who achieved freedom from MBS at 2 h, 62.8% (839/1335) were also pain free; this finding contrasts with 29.1% (388/1335) with mild pain and 8.1% (108/1335) with continued moderate/severe pain (Table 3). Similarly, in patients who experienced freedom from functional disability at 2 h post-dose, the proportion of patients who were also pain free was higher (86.1%, 795/923) than the proportion of patients who improved to mild pain (12.6%, 116/923) (Table 3). While lasmiditan-treated patients were more likely to achieve pain freedom than placebo-treated patients, among those who were pain free, the portions with successful outcomes on other endpoints were similar between patients who received lasmiditan versus placebo (Table 4).

**Table 4:** Proportion of patients with additional outcomes at 2 h by treatment and head pain groups

	Placebo			Lasmiditan 50 mg			Lasmiditan 100 mg			Lasmiditan 200 mg		
	PF	MP	CM/S	PF	MP	CM/S	PF	MP	CM/S	PF	MP	CM/S
Outcome(s)	***N*** = 180	***N*** = 239	***N*** = 414	***N*** = 135	***N*** = 138	***N*** = 160	***N*** = 274	***N*** = 267	***N*** = 243	***N*** = 324	***N*** = 220	***N*** = 235
MBS freedom, n (%)	168 (93.3)	98 (41.0)*	42 (10.1)*	126 (93.3)	60 (43.5)*	19 (11.9)*	249 (90.9)	131 (49.1)*	23 (9.5)*	296 (91.4)	99 (45.0)*	24 (10.2)*
Functional disability freedom^a^, n (%)	166 (92.2)	34 (14.2)*	3 (0.7)	124 (91.9)	20 (14.5)*	1 (0.6)	240 (87.6)	34 (12.7)*	4 (1.6)	265 (81.8)	28 (12.7)*	4 (1.7)
PGIC (much better/very much better), n (%)	159 (88.3)	65 (27.2)*	7 (1.7)	117 (86.7)	52 (37.7)*	2 (1.3)	243 (88.7)	84 (31.5)*	5 (2.1)	271 (83.6)	71 (32.3)*	4 (1.7)
Both MBS freedom and disability free^a^, n (%)	161 (89.4)	27 (11.3)*	1 (0.2)	119 (88.1)	16 (11.6)*	0 (0.0)	228 (83.2)	28 (10.5)*	2 (0.8)	255 (78.7)	22 (10.0)*	0 (0.0)
MBS freedom, disability freedom^a^, and PGIC (much better/very much better), n (%)	145 (80.6)	16 (6.7)*	1 (0.2)	107 (79.3)	13 (9.4)*	0 (0.0)	208 (75.9)	20 (7.5)*	1 (0.4)	223 (68.8)	15 (6.8)*	0 (0.0)

*p < 0.001 vs group with pain freedom at 2 h. *P*-values were only calculated from odds ratios if the number of patients in each specific category was ≥10

^a^Functional disability was assessed with the question “How much is your migraine interfering with your normal activities?”. Response options were “not at all”, “mild interference”, “marked interference”, or “need complete bed rest”. Functional disability freedom was defined as having “Not at all” recorded at 2 h. Patients who recorded “Not at all” at the time of dosing were excluded from the analysis

Notes: *p*-values were generated from a two-sided test from a logistic regression model with study, head pain outcome group, and background use of medication to reduce the frequency of migraine as covariates. Firth’s penalized likelihood approach was used to address issues of quasi-complete separation

CM/S: continued moderate/severe pain; MBS: most bothersome symptom; MP: mild pain; PF: pain freedom; PGIC: patient global impression of change

We then evaluated the odds of achieving 2-h MBS freedom or functional disability freedom by head pain outcome groups. Pain outcomes (pain free or mild pain) were independently paired with MBS and disability outcomes. Patients who were pain free at 2 h were more likely to be MBS free at 2 h in comparison with those who did not achieve pain freedom (patients with mild, moderate, or severe pain) (odds ratio = 35.5). Patients who had mild pain were more likely to be MBS free than those who continued to have moderate/severe pain (odds ratio = 7.1). Patients who were pain free at 2 h were more likely to be free of functional disability at 2 h in comparison with those who did not achieve pain freedom (odds ratio = 64.0). Patients who experienced mild pain were also more likely to be free of functional disability than those who continued to have moderate/severe pain (odds ratio = 13.4).

### Concurrence of pain outcomes with freedom from MBS and functional disability

**Fig. 1:**
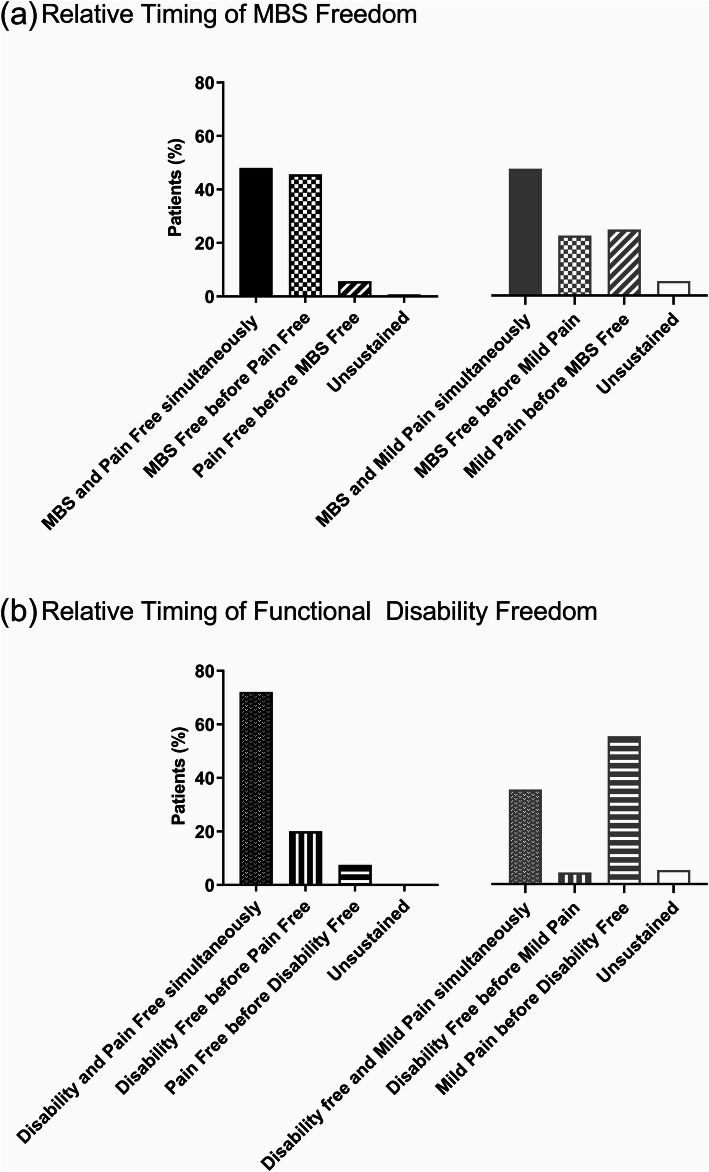
Relative timing of freedom from most bothersome symptom (MBS) or functional disability and pain status. (**a**) Sequence of outcomes in patients that experienced MBS freedom and either pain freedom (*N* = 839) or mild pain (*N* = 388) at 2 h. (**b**) Sequence of outcomes in patients that experienced functional disability freedom and either pain freedom (*N* = 795) or mild pain (*N* = 116) at 2 h. Notes: Unsustained represented patients who experienced freedom from MBS or functional disability freedom and either freedom from pain or improvement to mild and then moved out of that group prior to 2 h post-dose. The denominator was the total population that achieved the MBS freedom or disability freedom at 2 h post-dose in either the pain free or mild pain groups

We next explored the relative timing of achievement of outcomes for patients who achieved freedom from pain or mild pain with either freedom from MBS or functional disability at 2 h. The proportions of patients experiencing the outcomes sequentially or both outcomes at the same assessment time point are shown in Fig. 1. The proportions of patients achieving freedom from MBS and pain freedom or mild pain concurrently were similar (48.0% for freedom from MBS with freedom from pain and 47.4% for freedom from MBS with mild pain) (Fig. 1a). In contrast, the majority of patients experienced freedom from functional disability concurrently with freedom from pain (72.1% for pain free vs 35.3% with mild pain) (Fig. 1b). This result was similar regardless of treatment (Supplementary Fig. 1). Therefore, freedom from functional disability was more frequently concordant with freedom from pain than improvement to mild pain, and this was independent of treatment.

## Discussion

This project assessed the association between freedom from pain or improvement to mild pain with freedom from MBS, freedom from migraine-related functional disability, and overall improvement in a series of post hoc analyses. The main finding was that MBS freedom, functional disability freedom, and improved PGIC were reported significantly more frequently in patients who achieved pain freedom compared to those who reported mild pain. The proportion of patients who experienced all three positive outcomes was approximately ten times higher in patients who achieved pain freedom compared to those with improvement to mild pain. Although pain free rates were higher with lasmiditan than placebo, among those who achieved pain freedom, rates of MBS freedom, functional disability freedom, and improved PGIC were similar across treatment groups. These findings suggest the relationships of pain status to other outcomes at 2 h is independent of treatment with lasmiditan.

In this study, only 8% of patients achieving pain freedom at 2 h did not achieve MBS freedom. Besides this high concordance between 2-h pain freedom and MBS freedom, inclusion of a measure of associated symptoms in clinical studies in general adds value because migraine is a symptom complex [7] that includes pain and associated symptoms. We believe that treatments for migraine should relieve both pain and the associated symptoms that patients find bothersome.

Functional disability freedom and pain freedom occurred simultaneously in a high proportion of patients, suggesting a close temporal association of these outcomes. The analysis showed that in the subset of patients with pain freedom and functional disability freedom by 2 h, the majority (72.1%) achieved both outcomes at the same assessment time. These findings indicate that the outcome of pain freedom generally occurs with return to normal function. Similar results were observed in the placebo group, which implies that the observation may be related to disease recovery, whether spontaneous or induced by treatment.

Previous studies [8–13] evaluated qualities most important to patients when assessing the efficacy of an acute treatment of migraine. Rapid onset of complete pain elimination is a priority for patients and is a predictor of satisfaction with acute therapy [11]. Pain relief, in contrast, especially at early time points, consists mainly of reduction to mild head pain rather than pain freedom. Based on analysis of randomly assigned, controlled trials of acute treatments for migraine, including lasmiditan, aspirin, ibuprofen, ergotamine, and triptans (sumatriptan, zolmitriptan, naratriptan, almotriptan, and rizatriptan), mild headache constitutes 90% of patients with headache relief after 0.5 h versus 40% of patients with headache relief at 2 h [14]. Furthermore, among patients with episodic migraine, insufficient acute treatment effectiveness is associated with a higher risk of new onset chronic migraine within 1 year [15], and pain freedom was associated with a lower likelihood of and delayed headache recurrence in a study of naratriptan [16]. This current analysis revealed that in comparison with 2-h mild pain, patients achieving 2-h pain freedom were more likely to also achieve MBS freedom, return to normal function, and improved PGIC. Thus, these findings provide additional support for the use of head pain freedom at 2 h as a primary outcome measure of efficacy for acute treatments of migraine.

One analysis suggested that pain relief was associated with decreased migraine-associated disability and that functional ability can be restored before pain freedom [17]. However, pain relief in that study included those with both mild pain and no pain 2 h post-dose. In our analysis, we found that freedom from functional disability was reported before the onset of pain freedom in 20% of patients. Rates of freedom from functional disability in those with mild pain at 2 h are modest (13.4%) compared to those with freedom from pain at 2 h (87.1%). Moreover, 72.1% of patients achieved freedom from pain and disability concurrently versus 35.3% with mild pain.

Strengths of this post hoc analysis included the large number of patients pooled from two almost identical Phase 3 clinical trials. The results show a large difference between patients achieving pain freedom versus those reporting mild pain that is biologically plausible and unlikely to be a result of chance. The findings were consistent regardless of treatment assignment to active drug versus placebo in these clinical trials.

A limitation is that it is unknown whether other acute treatments for migraine could potentially alter the relationships among study outcomes or if similar results would be obtained with other treatments. In addition, this analysis examined three levels of pain outcomes (none, mild, or moderate/severe). Certain baseline characteristics were not evenly distributed among the three groups, and it is possible that the migraine attacks experienced in the continued moderate/severe pain group are more difficult to treat than the other groups. Several studies have shown that early dosing was associated with mild pain [18, 19]. As more patients in the 2-h pain free group vs the improved to mild pain group had severe pain at baseline, any benefit of early time to dosing observed in this study becomes less clear. Additionally, time to dosing with lasmiditan does not greatly impact the proportion of patients experiencing pain freedom [20]. Although functional status was evaluated on a 4-point ordinal scale, we evaluated functional status as a binary outcome (disability freedom or no disability freedom) because disability freedom is the therapeutic goal of treatment. In a previous analysis of 2 randomized, placebo-controlled clinical trials of rizatriptan, complete satisfaction with treatment was more common in patients returning to normal function (23 and 11%) versus those with mild impairment (1.3 and 1.6%) at 2 h [9].

## Conclusion

Compared with improvement to mild pain, freedom from head pain at 2 h post-dose was more frequently associated with freedom from MBS, freedom from migraine-related functional disability, and improved PGIC at 2 h post-dose. Thus, in the acute treatment of migraine, freedom from pain appears to be a more powerful predictor of other clinically important outcomes than improvement to mild pain. Pain freedom is a highly desirable outcome for patients and is a valid endpoint to indicate the effectiveness of an acute treatment for migraine.

## Supplementary Information

**Additional file 1: Supplemental Figure 1.** Relative timing of freedom from functional disability and pain status by dose. Sequence of outcomes in patients that experienced functional disability freedom and either pain freedom (left panel) or mild pain (right panel) at 2 h. Notes: “Unsustained” category represented patients who experienced freedom from MBS or functional disability freedom and either freedom from pain or improvement to mild and then moved out of that group prior to 2 h post-dose. The denominator was the total population that achieved the MBS freedom or disability freedom at 2 h post-dose in either the pain free or mild pain groups.
