# Supplementary figures and images for: A close association of freedom from pain, migraine-related functional disability, and other outcomes: results of a post hoc analysis of randomized lasmiditan studies SAMURAI and SPARTAN
Source: J Headache Pain. 2021 Aug 28;22(1):101. doi: 10.1186/s10194-021-01303-w (PMC8400846; doi:10.1186/s10194-021-01303-w)

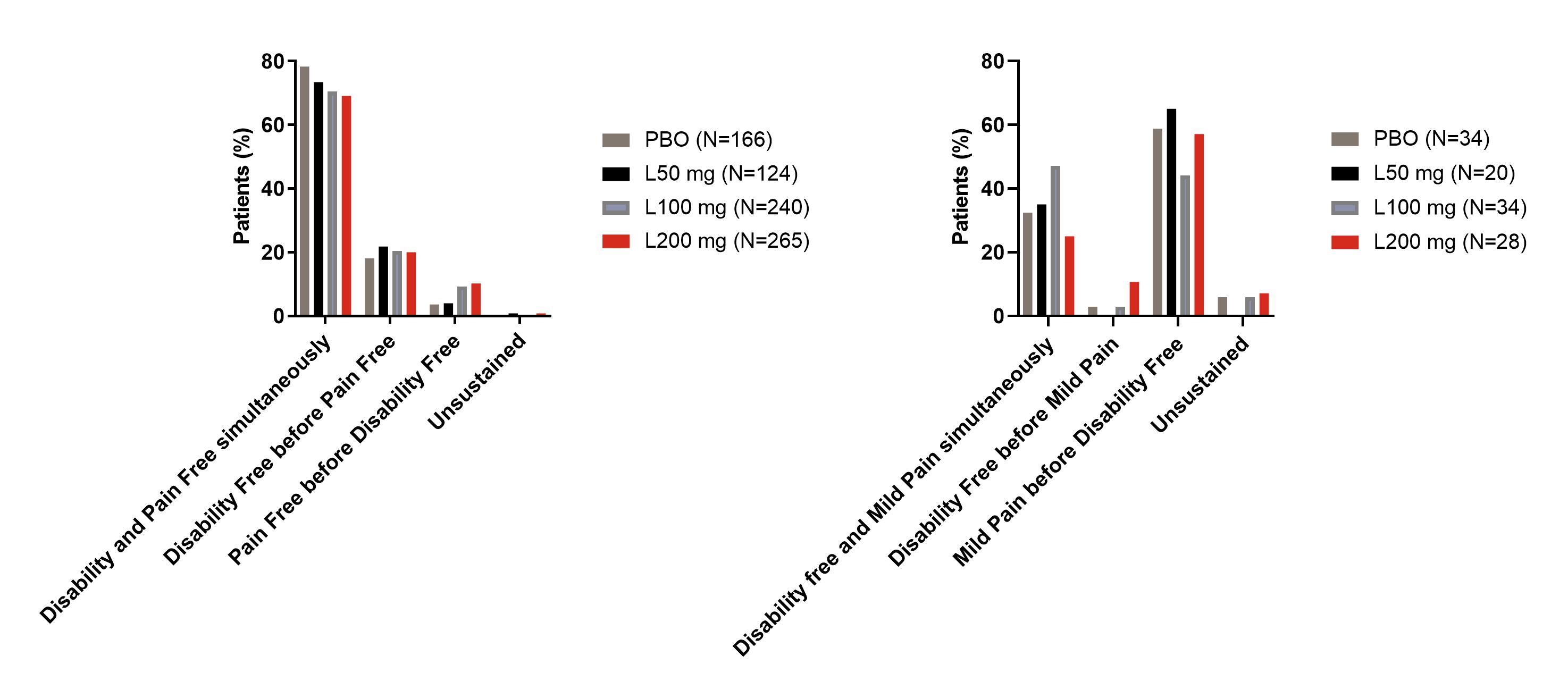

Supplement: Supplementary file 1 — Additional file 1: Supplemental Figure 1. Relative timing of freedom from functional disability and pain status by dose. Sequence of outcomes in patients that experienced functional disability freedom and either pain freedom (left panel) or mild pain (right panel) at 2 h. Notes: “Unsustained” category represented patients who experienced freedom from MBS or functional disability freedom and either freedom from pain or improvement to mild and then moved out of that group prior to 2 h post-dose. The denominator was the total population that achieved the MBS freedom or disability freedom at 2 h post-dose in either the pain free or mild pain groups. [file 10194_2021_1303_MOESM1_ESM.jpg]
